# Supplementary material for: Therapeutic Potential of Bromodomain and Extra-Terminal Domain Inhibitors for Synovial Sarcoma Cells
Source: Cancers (Basel). 2024 Mar 11;16(6):1125. doi: 10.3390/cancers16061125 (PMC10968860; doi:10.3390/cancers16061125)

Figure 1C.

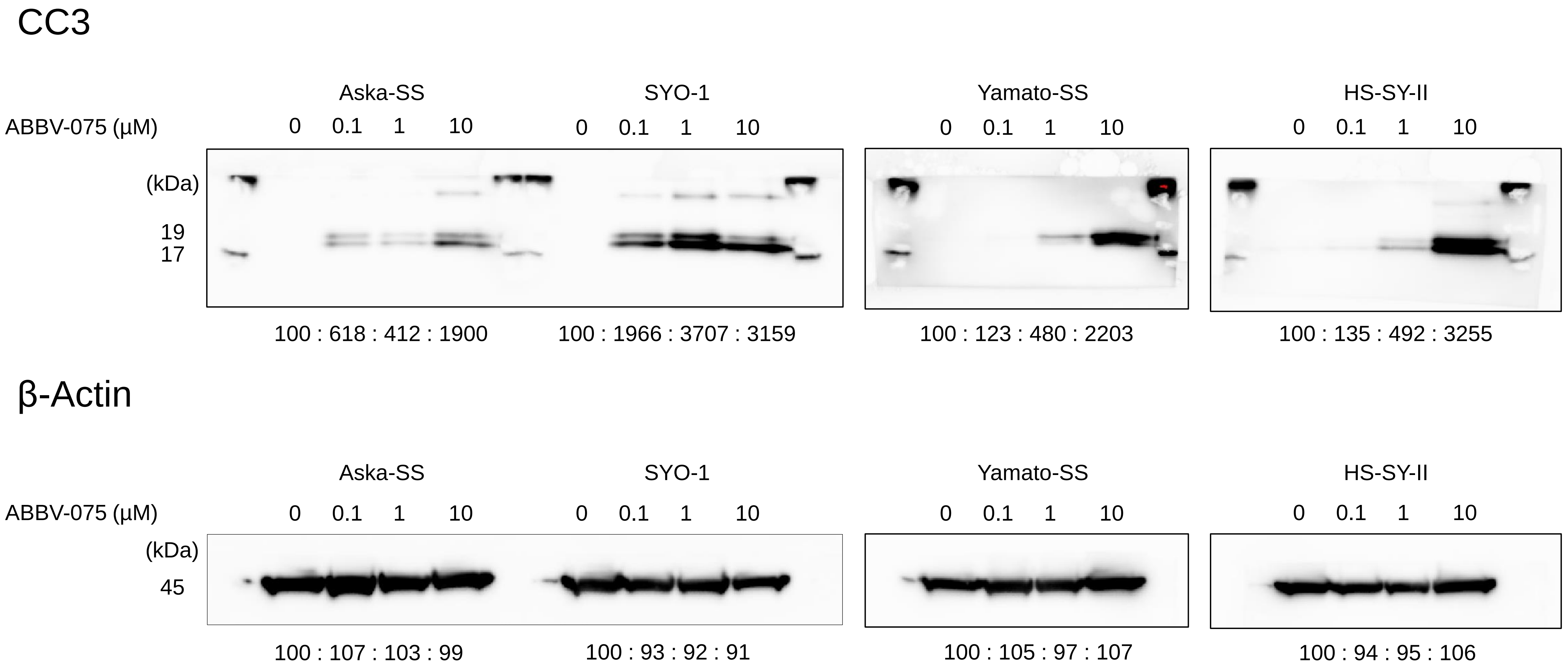
CC3
Aska-SS
SYO-1
Yamato-SS
HS-SY-II
ABBV-075 (μM)
0 0.1 1 10
0 0.1 1 10
0 0.1 1 10
0 0.1 1 10
(kDa)
19
17
100 : 618 : 412 : 1900
100 : 1966 : 3707 : 3159
100 : 123 : 480 : 2203
100 : 135 : 492 : 3255
β-Actin
Aska-SS
SYO-1
Yamato-SS
HS-SY-II
ABBV-075 (μM)
0 0.1 1 10
0 0.1 1 10
0 0.1 1 10
0 0.1 1 10
(kDa)
45
100 : 107 : 103 : 99
100 : 93 : 92 : 91
100 : 105 : 97 : 107
100 : 94 : 95 : 106

Figure 3B.

MYC

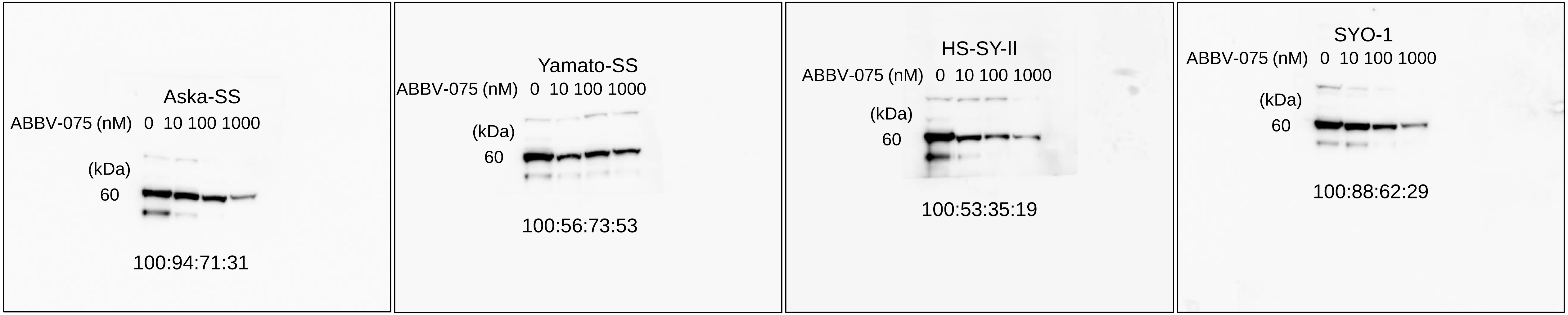

p53

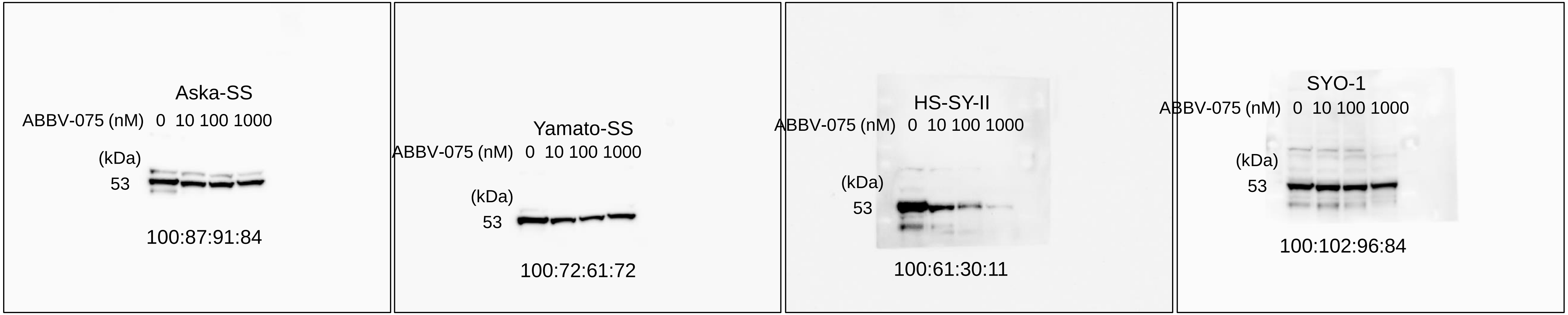

Figure 3B.

p21

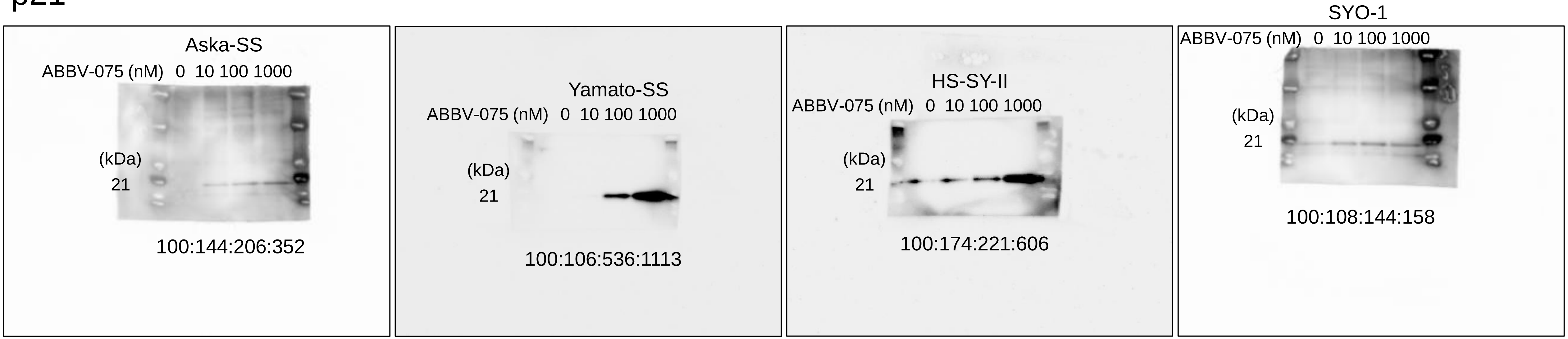

CCND1

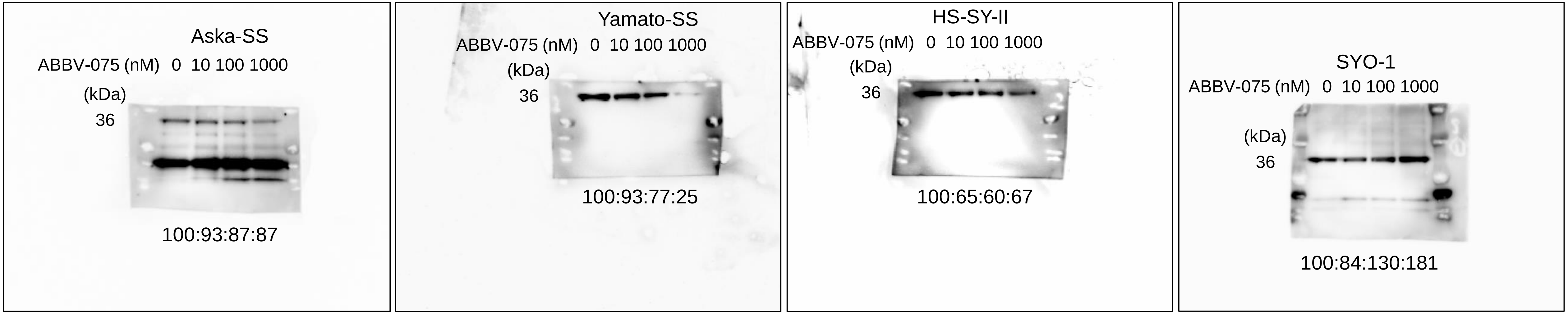

Figure 3B.

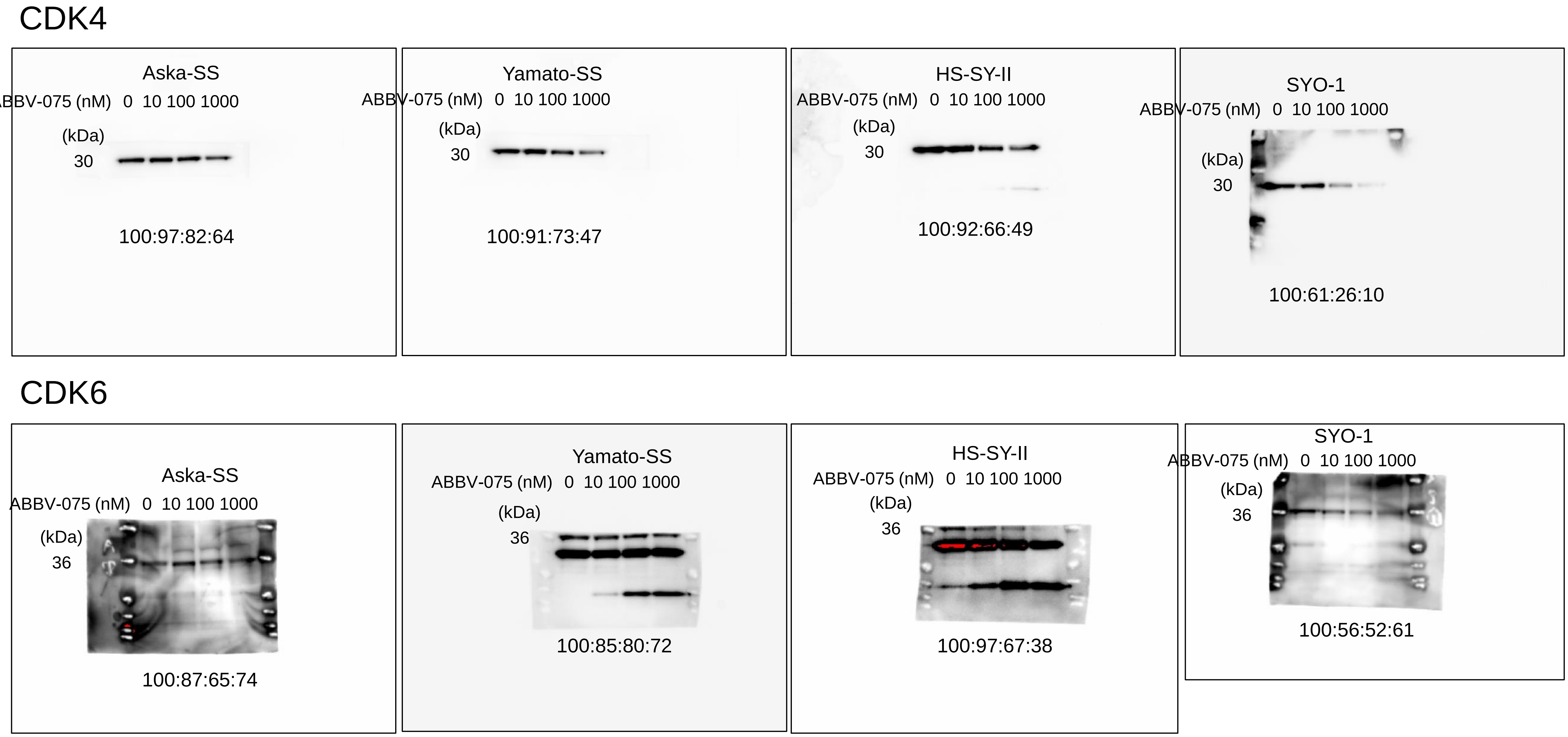
CDK4
Aska-SS
ABBV-075 (nM) 0 10 100 1000
(kDa)
30
100:97:82:64
Yamato-SS
ABBV-075 (nM) 0 10 100 1000
(kDa)
30
100:91:73:47
HS-SY-II
ABBV-075 (nM) 0 10 100 1000
(kDa)
30
100:92:66:49
SYO-1
ABBV-075 (nM) 0 10 100 1000
(kDa)
30
100:61:26:10
CDK6
Aska-SS
ABBV-075 (nM) 0 10 100 1000
(kDa)
36
100:87:65:74
Yamato-SS
ABBV-075 (nM) 0 10 100 1000
(kDa)
36
100:85:80:72
HS-SY-II
ABBV-075 (nM) 0 10 100 1000
(kDa)
36
100:97:67:38
SYO-1
ABBV-075 (nM) 0 10 100 1000
(kDa)
36
100:56:52:61

Figure 3B.

β-Actin

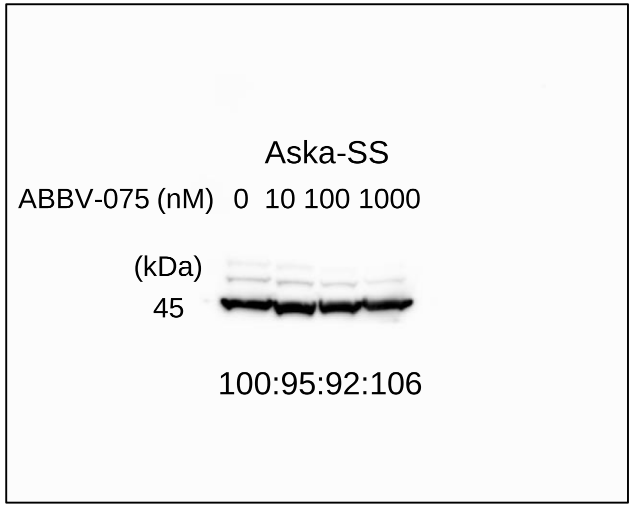

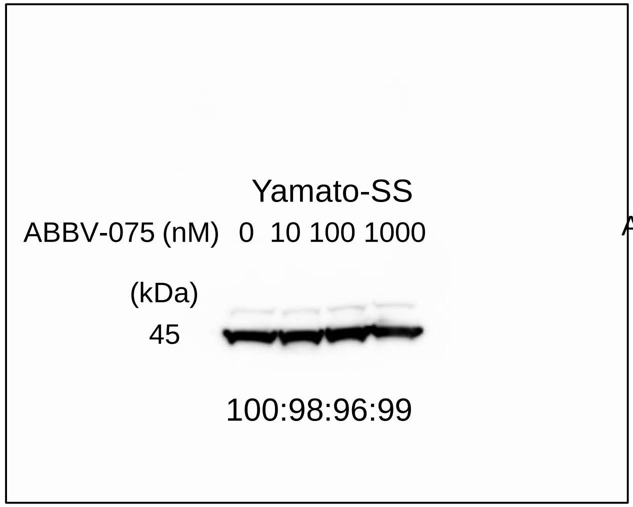

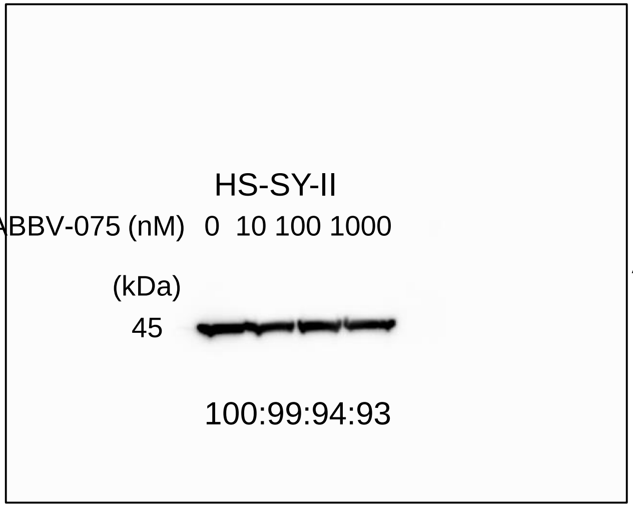

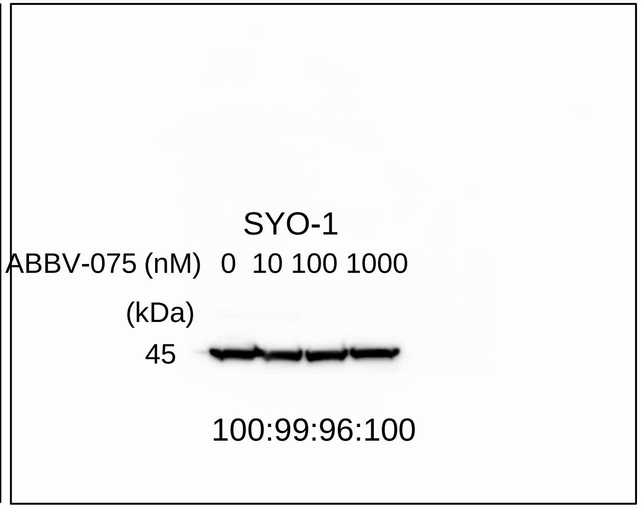

Figure 4D.

BCL2

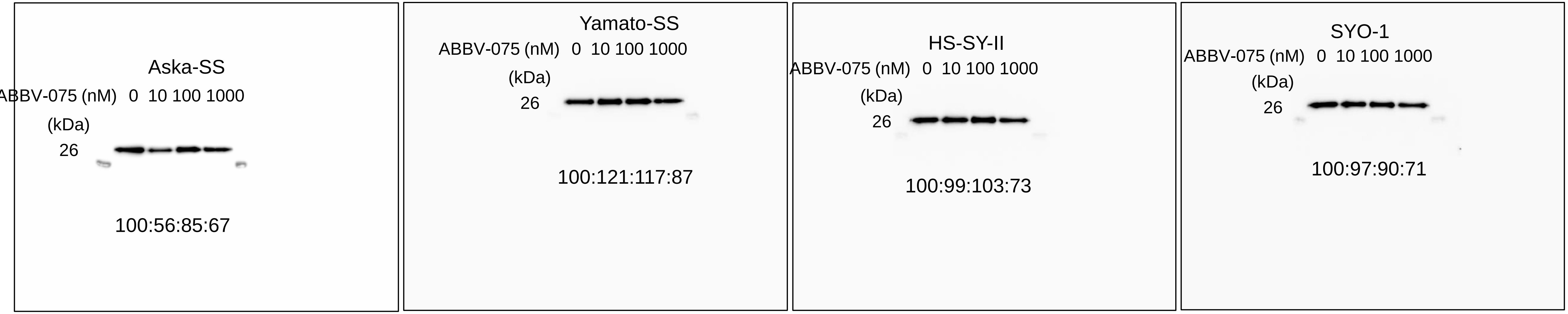

BCLxL

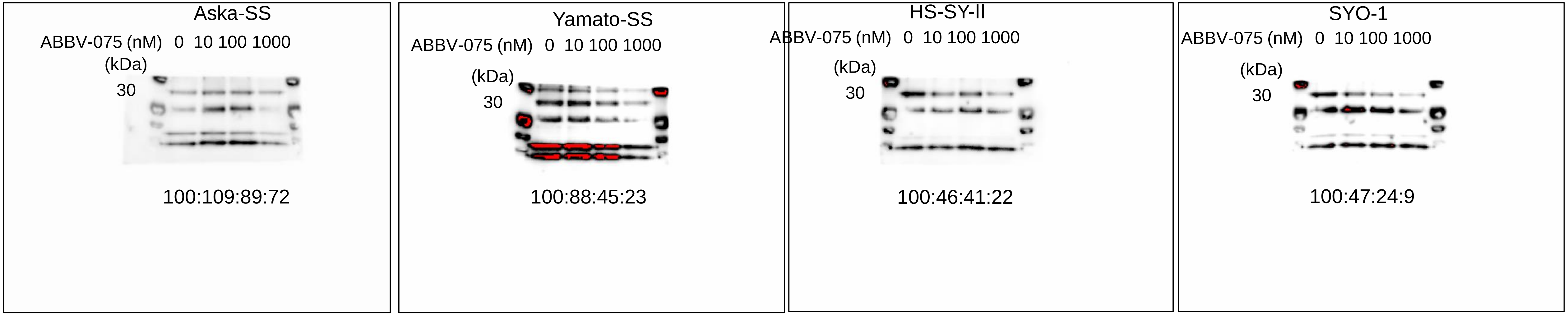

Figure 4D.

BIM

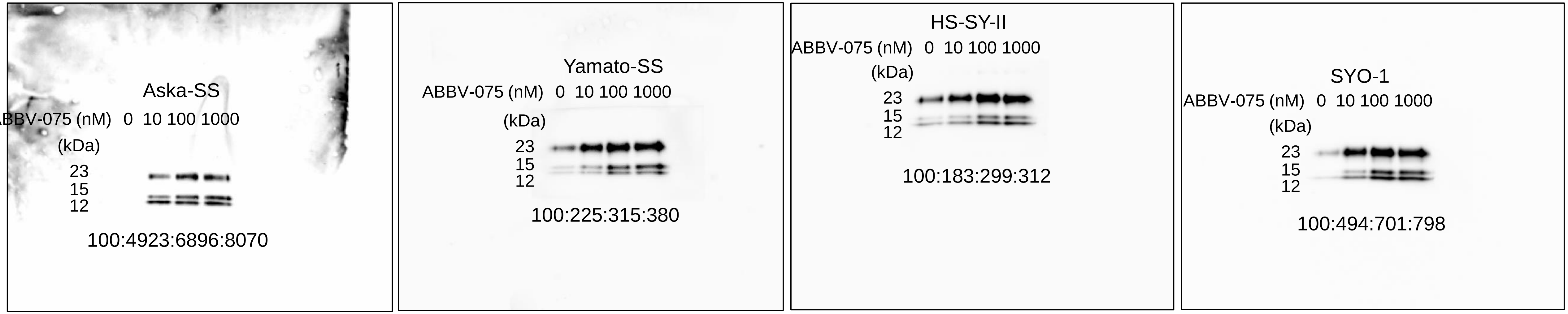

PUMA

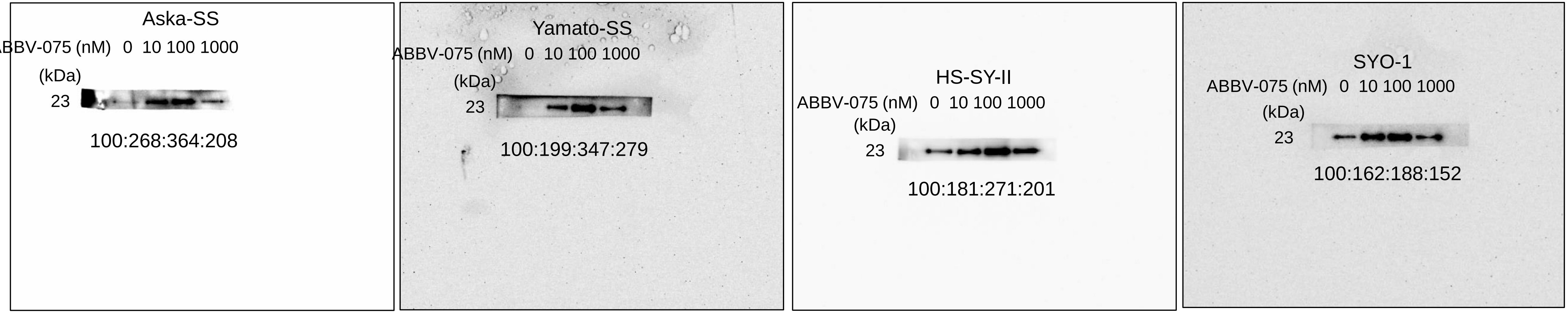

Figure 4D.

β-Action

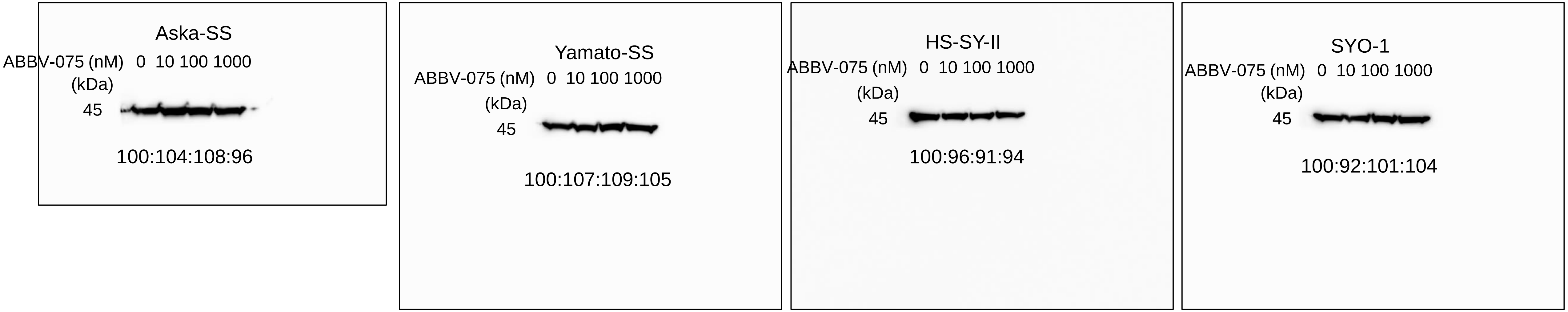

Figure 5E.

SS18-SSX

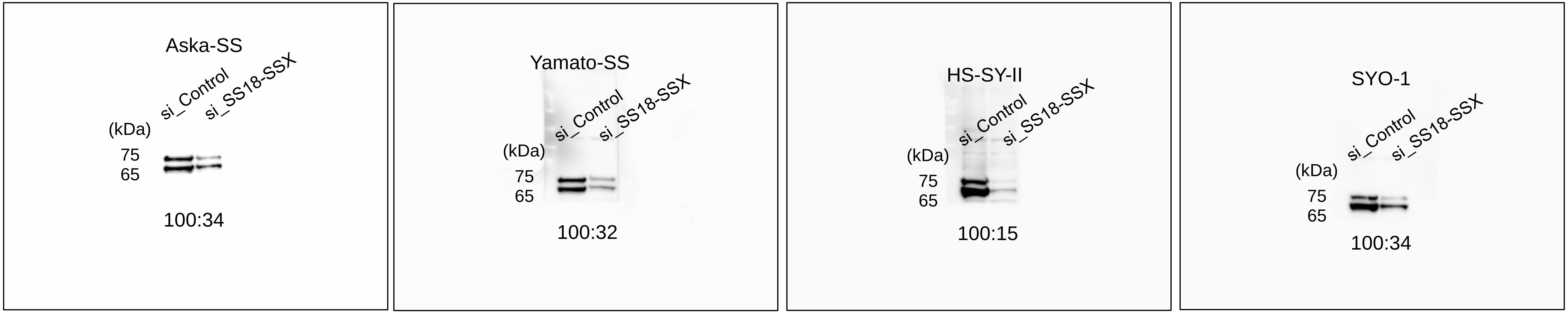

BCL2

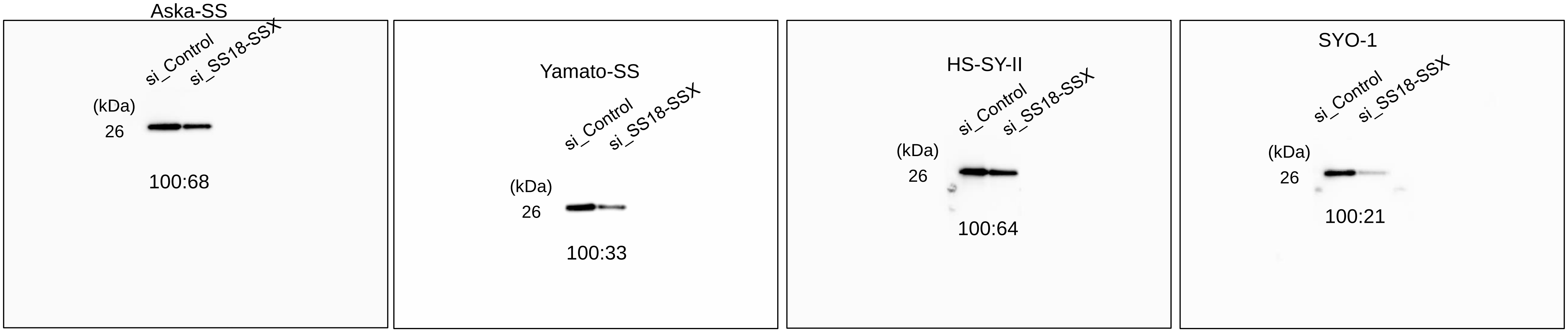

Figure 5E.

BCLxL

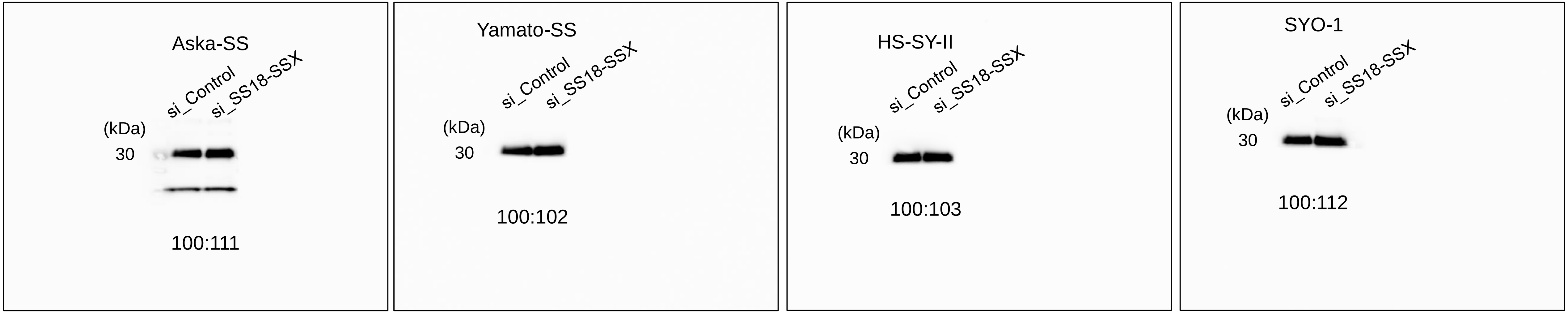

BIM

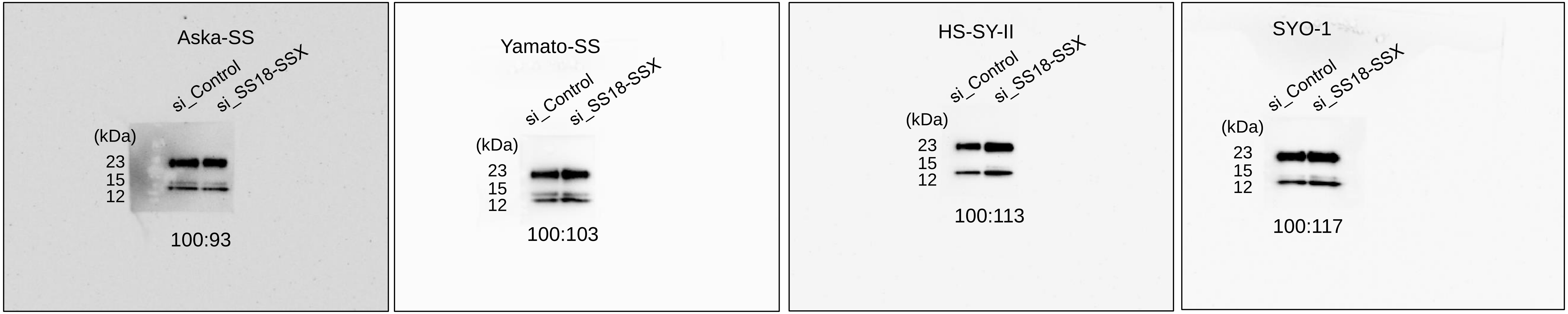

Figure 5E.

β-Actin

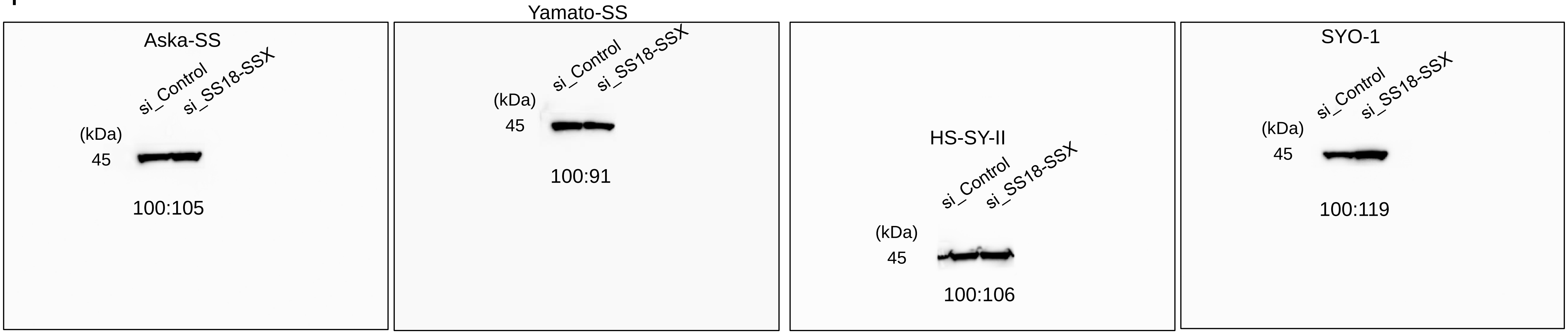

Supplement: Supplementary file 1 [file cancers-16-01125-s001.zip › 20240224_Supplementary File S1.pdf]
