# Supplementary material for: Therapeutic Potential of Bromodomain and Extra-Terminal Domain Inhibitors for Synovial Sarcoma Cells
Source: Cancers (Basel). 2024 Mar 11;16(6):1125. doi: 10.3390/cancers16061125 (PMC10968860; doi:10.3390/cancers16061125)
Supplement: Supplementary file 1 [file cancers-16-01125-s001.zip › 20240307_Supplementary Information.pdf]

Supplementary Information

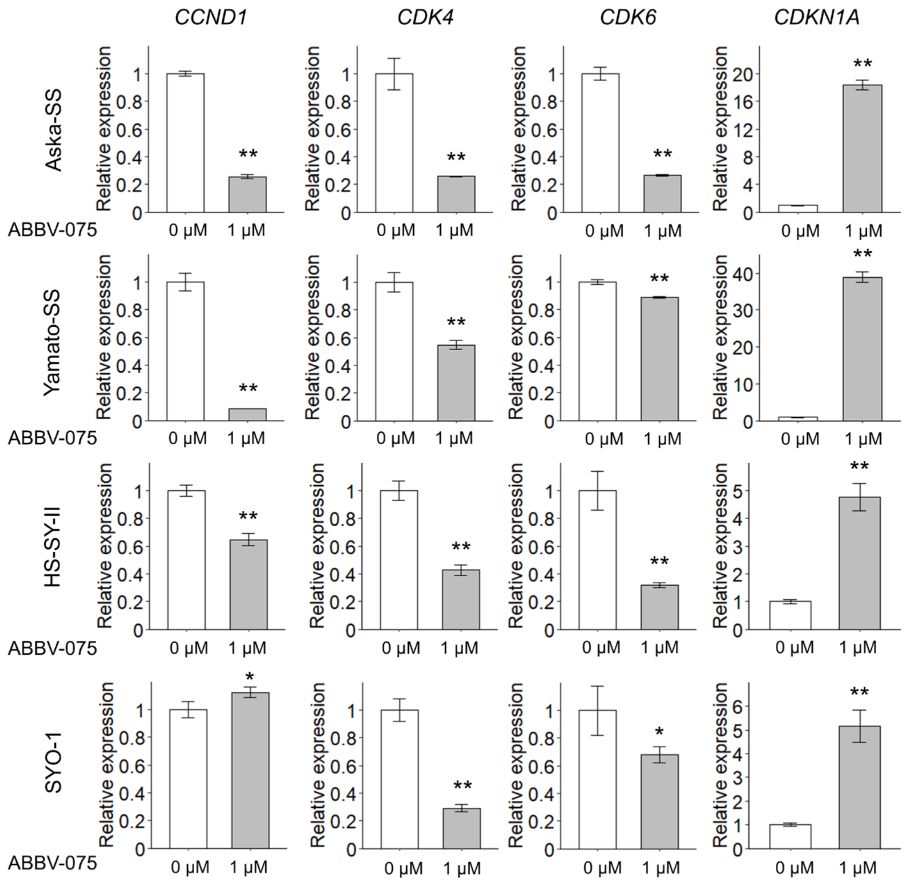

Supplementary Figure S1. mRNA expressions of cell cycle regulators, CCND1, CDK4, CDK6, and CDKN1A, of four synovial sarcoma cell lines treated with 0 μM and 1 μM of ABBV-075 examined using quantitative polymerase chain reaction. Data are represented as means ± standard deviation. *, P < 0.05; **, P < 0.01 (Student's *t*-test).

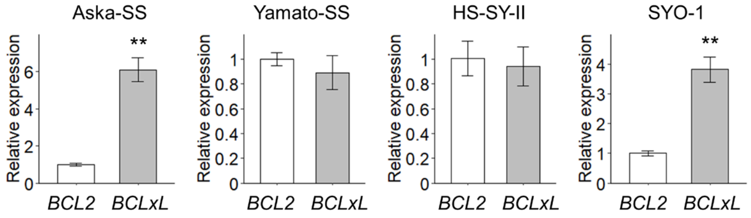

Supplementary Figure S2. mRNA expressions of BCLxL compared with BCL2 in four synovial sarcoma cell lines examined using quantitative polymerase chain reaction. Data are represented as means ± standard deviation. **, P < 0.01 (Student's *t*-test).
